# 

**Published:** 1887-04-30

**Authors:** 


### The Hospital.

**An Institution, Family, and Parochial Journal of Hospitals, Asylums, and all Agencies for the Care of the Sick, Criticism and News.**

SATURDAY, APRIL 30TH, 1887.

### Hospital Housekeeping.

Forms and full particulars of the Quilter Prizes under this head (see page 355 for 19th Feb.) may be had on application to the Editor of The Hospital, Norfolk House, W.C. All competitive essays to be sent in by 30th April, 1887. Prizes, £10 10*s.* and £5 5*s.*

### The Literary Prize.

A prize of **Two Guineas** will be given every month for the best story or incident based upon hospital, or asylum, or student life, or any incident connected with the professions of medicine or nursing.

### THE CHILDREN'S CORNER.

**Third Quarterly Prize Competition.**
Began 2nd Apr., 1887 ; ends 25th June, 1887.

PRIZES.
FOR MOST CORRECT ANSWERS TO PUZZLES.

| | | | |
|---|---|---|---|
| 1st .. | .. Thirty Shillings | 3rd .. | .. Ten Shillings |
| 2nd .. | .. Twenty „ | 4th .. | .. Five „ |

FOR NEATEST ANSWERS TO PUZZLES.
Five Shillings.
FOR BEST LITTLE LETTERS.
Ten Shillings.

**Puzzles—Fifth Week.**

No. 20. CONUNDRUM. Why is an old hen walking towards Whitehall like the Gunpowder Plot?

No. 21. ENIGMA. A man had to cross a river and carry with him a goose, a fox, and a bag of barley, and he was allowed to take only one at a time. How could he do so, and at the same time safely leave two of them together, for the fox would eat the goose, and the goose would eat the barley?

No. 22. CHARADE.

My *first's* the joy of ev'ry cozy dame,
And in my *second* o'er to England came ;
My *whole* of ev'ry household forms a part ;
Thou art not Science, but thou teachest Art.

No. 23. BURIED AUTHORS. Trembles and a long pointed weapon ; a covering for the head ; injuries caused by fire ; a boy's Christian name and a male descendant ; the comparative of much ; a fastening for a door.

**Letters—Fifth Week.**

The Letter-subject for next week will be "A Country Ramble."

**Results of Third Week—Puzzles.**

No. 10. CONUNDRUM. Heat travels faster, because you can *catch* cold. Some other clever answers have been given.

No. 11. DECAPITATION. Chart, hart, char.

No. 12. FRENCH PUZZLE. Oiseau.

No. 13. PUZZLE SENTENCE. His illness lasted from the *beginning* of *A*pril till the *end* of Jun*e*.

No. 14. DROPPED LETTER PUZZLE.

One thing at a time,
And that done well,
Is a very good rule,
As many can tell.

*All right*—A. C. K., Agatha, Tim, Umslopogaas, Scrooge ; *Four right*—Bohemian Girl, Dot, R. Epton, Joe, A. G. S. McCulloch, Mikado, Nordisa, Skye, Gom ; *Three right*—Ellie, Etta ; *Two right*—Peeler.

**Letters.**

Most of those "Easter holiday" letters were, to borrow the phraseology of one little girl respecting her trip, "quite delightful." At any rate their perusal affords me much pleasure. Etta, who is only just turned eight, writes her first letter to me, and a very nice one it is. Scrooge spent her Easter holidays at Blackpool, where the weather was lovely. Mikado and her sister, Bohemian Girl, both write excellent letters. Skye went to Chatham Hill, and gives me an admirable account of a very large house now in course of erection there by a peculiar sect who style themselves the Jesraelites. It is, she says, like a temple, and is to last till judgment day! Ellie's little brother is ill, so that her Easter holidays were not quite so enjoyable as they would otherwise have been.

Here is a letter from a new friend whom I am very pleased to welcome :—

DEAR MR. EDITOR,—Holidays are, as a rule, pleasant to the young, especially when they come in such fine weather as we have been having lately. My Easter holidays have, on the whole, been very enjoyable, although I must own to a little disappointment. I spent them all near home. On Saturday I took a walk to Christleton, and visited the church, which was decorated very nicely for Easter Day. On Monday I had an enjoyable treat up the river, which was crowded with visitors from various places. I walked around Eaton Hall and its grounds, to the Iron Bridge, from thence to Eccleston Ferry, returning to the Groves by steamer.

I am, dear Mr. Editor, yours truly, ADA, aged 12.
6, Bold Place, Chester.

Agatha writes me the following interesting letter :—

DEAR MR. EDITOR,—On Easter Eve we helped to decorate the church, and I helped to put the daffodils and moss in the windows. I played cricket every day in Easter week, which I am very fond of. On Wednesday, a gentleman here had a paper-chase, and I ran in it nearly eight miles without stopping, over hills, and through valleys and woods; four times we waded through brooks ; and I came in first of the children. On Friday the same gentleman gave us some athletic sports, and I won the long jump. The obstacle race was great fun ; the tarpaulin was very rotten, and kept tearing away from the posts as we went under it. In the men's obstacle race, the tarpaulin posts gave way when the men were inside, and they all rolled down the hill.

I remain, yours truly, AGATHA, aged 10½.

Porlock, Taunton.

One mark each:—Ellie, Skye, Scrooge, Etta, Ada, Agatha, Bohemian Girl, Tim, Mikado.

**Rules.**

Only one side of the paper should be written upon.

The address and full Christian name and surname, and the age of the competitor, must be stated in all replies. A *nom de plume* may be added for the purposes of publication.

The first prize may not be taken by the same competitor more than once in any one year. In the event of the winner of the first prize again scoring highest, the second prize will be awarded, the next competitor receiving the first.

Answers must be addressed to the "PUZZLE EDITOR," THE HOSPITAL, Norfolk House, Norfolk Street, Strand, W.C., not later than Thursday next. The "Children's Corner" Coupon (see advertisement pages) must be enclosed.

### AMUSEMENTS WITH PROFIT.

### PRIZE COMPETITIONS.

**Quarterly Prize Competition.**
Began 23rd April ; ends 16th July.

**A Prize of Three Guineas**

will be awarded to the Competitor who shall have sent in the largest number of correct or best answers. No one will be permitted to win the Three Guinea Prize twice in any one year, but we shall award annually an additional

**Prize of Five Guineas**

to the competitor who shall have sent in the largest number of correct or best answers during the twelve months.

**No. 3. Double Acrostic.**

A Children's Hospital you'll see,
When all my lights you have set free.
* * * * * * *
A *musical genius*, composer far fam'd ;
In Guyana a *stream* by Dutchmen nam'd ;
The *son of Eunomus*, to Sparta gave laws ;
A *tattler* who talks without reason or cause ;
This great *painter-poet* has now pass'd away ;
The son of Duke Robert was killed at this *fray* ;
A *town* for its bread and its beer renowned,
This last *'twixt our isle and France* will be found.

**No. 4.—Mathematical Question.**

On 21st June 1851, a man had lived just 30,000 days. Find the day and year of his birth.

**Answers.**

No. 25. DOUBLE ACROSTIC.

D ionysiu S

O rmol U

C entau R

T hu G

O ud E

R avenspu R

S avo Y

Correct answers have been received from K. M., A. M. E., Ostrich, May, Mater, Boss, Ellice, Mrs. Bedingfeld, Peeler, Gretta.

No. 26. MATHEMATICAL QUESTION.

A little consideration will easily lead one to the solution without the application of any rule, but, solved as a question in Compound Proportion, the terms will stand :

1½ mice : 100 mice :: 1½ cats : x

50 min. : 1½ min.

Answer—3 Cats.

Correct answers have been received from Ostrich, Mater, Perseverance, Ellice.

**Results of First Prize Competition.**

Honours thus far are divided, Ostrich and Ellice each scoring twenty-five correct answers. Under these circumstances we propose either (1) to divide the prize, leaving both competitors still free to enter again for the Three Guinea Prize ; or (2) to set a test acrostic to decide the tie. We shall be pleased to hear from Ostrich and Ellice which course they would like us to adopt.

Answers must be addressed to THE PRIZE EDITOR, AMUSEMENTS WITH PROFIT, THE HOSPITAL, Norfolk House, Norfolk Street, Strand, W.C., not later than the *first* post on Saturday next. The full name and address must in all cases be given, but a *nom de plume* may be added for publication. Only one side of the paper should be written on. The "Amusements with Profit" Coupon (see advertisement pages) must be enclosed with replies.

### THE IN- AND OUT-PATIENTS' HOSPITAL DIARY.

This Diary is so arranged as to make some portion of it appear every week, and the whole in each monthly part. It has been very difficult to compile, and corrections will at all times be gratefully received. It has been suggested that similar information about the larger Provincial and Scotch Hospitals would be useful to many readers. We should be glad to hear if this is felt to be the case.

### DISEASES AND IRREGULARITIES OF THE TEETH.—*continued.*

| Hospitals and Terms of Admission. | Physicians, Surgeons, and Medical Officers, with Days and Hours of Attendance. |
|---|---|
| New Hospital for Women, 222, Marylebone rd., W. | *Surgeon*, Mr. H. Apperley |
| North-West London Hosp., 18, Kentish Town Rd., N.W. | *Surgeon*, Mr. W. A. Maggs, F. 9 |
| Paddington Green Children's Hospital, Paddington | *Surgeon*, Mr. C. Vincent Cotterell, W. 9 |
| Royal Free Hospital, Gray's Inn Road, W.C. | *Surgeon*, Mr. H. Harris, M. Th. |
| Royal Hospital for Children and Women, Waterloo Bridge Road, S.E. | *Surgeon*, Mr. Whitehouse, F. 1.30 |
| St. Bartholomew's Hospital, Smithfield, E.C. | *Surgeons*, Messrs. Ewbank, Tu.; Paterson, F.; Ackery, F.; Mackrett, Tu. Hour, 9 |
| St. George's Hospital, Hyde Park Corner, S.W. | *Surgeon*, Mr. Winterbottom, Tu. S. 9 |
| St. Mary's Hospital, Cambridge Place, Paddington | *Susgeon*, Mr. H. Hayward, W. S. 9.30 |
| St. Thomas's Hospital, Westminster Bridge Road, S.E. | *Surgeon*, Mr. Truman, Tu. F. 10 |
| University College Hospital, Gower Street, W.C. | *Surgeon*, Mr. S. J. Hutchinson, W. 9.30 |
| Victoria Hospital for Children, Chelsea, S.W. | *Surgeon*, Mr. F. Fox, S. 9.0 |
| West London Hospital, Hammersmith Road, W. | *Surgeon*, Mr. H. L. Albert, Tu. F. 9.30 |
| Westminster Hospital, Broad Sanctuary, S.W. | *Surgeons*, Messrs. J. Walker and M. A. Smale, W. S. 9.15 |

### THROAT DISEASES.

| | |
|---|---|
| Central London Throat and Ear Hospital, Gray's Inn Road, W.C. | *Surgeons. See* "Ear Cases." M. W. Th. S. 2.30; Tu. F. 5 |
| Great Northern Central Hospital, Caledonian Rd, N. | *Surgeon*, Mr. Spencer Watson, W. 2 |
| Guy's Hospital, St. Thomas Street, S.E. | *Surgeon*, Mr. Symonds, Th. 1 |
| Hospital for Diseases of the Throat and Chest, 32, Golden Sq. Hon. Sec., Mr. G. C. Witherby. Poor free; others by small payment | *Physicians*, Drs. Wolfenden, Tu. F. 2.30; Macdonald, Tu. F. 6.30; Bond, W. S. 2.30. *Surgeon*, Mr. T. Mark Hovell, M. Th. 2.30 |
| King's College Hospital, Portugal Street, W.C. | *Surgeon*, Mr. Cartwright, Tu. F. 10 |
| Middlesex Hospital, Berners Street, W. | *Surgeon*, Mr. Hensman, Tu. 9 |
| North West London Hospital, 18, Kentish Tn. Rd., N.W | *Surgeon*, Mr. Collier, M. 1.30 |
| St. Bartholomew's Hospital, Smithfield, E.C. | *Surgeon*, Mr. Butlin, F. 2.30 |
| St. George's Hospital, Hyde Park Corner, S.W. | *Surgeon*, Dr. Whipham, Th. 1.30 |
| St. Mary's Hospital, Cambridge Place, Paddington | *Surgeon*, Mr. A. T. Norton, M. Th. 9.30 |
| St. Thomas's Hospital Westminster Bridge Road, S.E. | *Physician*, Dr. Semon, Tu. F. 1.30 |
| University College Hospital, Gower Street, W.C. | *Physician*, Dr. Poore, Th. 1.30 |
| Westminster Hospital, Broad Sanctuary, S.W. | W. 9. *See* General Hospitals. |

### DISEASES OF WOMEN, (OBSTETRIC CASES, ETC.)

| | |
|---|---|
| Charing Cross Hospital, West Strand, W.C. | *Physicians*, Drs. J. W. Black, Amand Routh, Tu. F. 1.30 |
| Chelsea Hospital for Women, Fulham Road, S.W. Sec., Mr. J. H. Easterbrook. Payment from 10*s*. 6*d*. a week. Poor by Governor's letter. Out-patients, if without subscriber's letter, pay 6*d*. each attendance | *In-Physicians*, Drs. J. H. Aveling, M. Th.; A. W. Edis, Tu. F.; F. Barnes, W. S. Hour, 2.30. *Out-Physicians*, Drs. Dickinson and Mackeron, M.; W. Travers and G. Harper, Tu.; F. Jones and J. I. Parsons, W.; and H. T. Rutherford, F. Hour, 2 |

### DISEASES OF WOMEN, (OBSTETRIC CASES, ETC.)—*Continued.*

| Hospitals and Terms of Admission. | Physicians, Surgeons, and Medical Officers, with Days and Hours of Attendance. |
|---|---|
| East London Hospital for Children and Dispensary for Women, Shadwell, E. A Subscriber's letter is required, except in urgent cases | *Out-Physicians*, M. Tu. W. Th. F. 1 and 3; Tu. F. 9. *See* "Children's Diseases." |
| German Hospital, Dalston Lane, Dalston, E. | *Physician*, Dr. Rasch, M. Th. 9 |
| Great Northern Central Hospital, Caledonian Rd., N. | *Physician*, Dr. F. Barnes, W. 2 |
| Grosvenor Hospital for Women and Children, 3 and 4, Vincent Square, S.W. | *Out-Physicians and Surgeons*, Tu. W. Th. S. 2. *See* "Children's Diseases." |
| Guy's Hospital, St. Thomas Street, S.E. | *Physicians*, Drs. Galabin, Horrocks, M. Tu. F. 1.30 |
| Hospital of St. John and St. Elizabeth, 47, Gt. Ormond Street, W.C. For females suffering from long standing disease. | *Physicians*, Drs. Constable and Tegart (no out-patients) |
| Hospital for Women, 29 and 30, Soho Sq., W. Sec., Mr. David Cannon. In-patients admitted free by Governor's letter; also by payment. Out-patients free | *In-Physicians*, Drs. Carter, W. S. 2; R. T. Smith, Tu. F. 2; E. Holland, Tu. F. 9.30. *In-Surgeon*, Mr. H. A. Reeves, M. 4, Th. 2; *Out-Physicians*, Drs. R. T. Smith, F. 10; E. Holland, W. 10; Mansell-Moullin, M. Th. 11; Bedford Fenwick, Tu. 10; J. Oliver, S. 10. *Out-Surgeon*, Mr. S. Osborn, Th. 2 |
| King's College Hospital, Portugal Street, W.C. | *Physician*, Dr. Playfair, Tu. Th. S. 2 |
| London Hospital, Whitechapel Road, E. | *In-Physician*, Dr. Herman, Tu. F. 1.30. *Out-Physician*, Dr. Lewers, W. S. 1.30 |
| Metropolitan Free Hospital, Gray's Inn Road, W.C. | *Physician*, Dr. A. Venn, W. 2 |
| Middlesex Hospital, Berners Street, W. | *In-Physician*, Dr. Edis, Tu. F. 1.30. *Out-Physician*, Dr. Duncan, W. S. 1.30 |
| New Hospital for Women, 222, Marylebone Road, W. Hon. Sec., Miss C. Vincent. By subscriber's letter. Outpatients pay 6*d*. entrance fee, and 2*d*. each visit. | *In-Physicians*, Drs. Anderson, Atkins, Marshall, de la Cherois. *Out-Physicians*, Drs. Marshall, De la Cherois, Hitchcock. Daily, 1 to 3, and S 9 to 10. (All the physicians are women) |
| North-West London Hospital, 18, Kentish Tn. Rd., N.W | W. 1.30. *See* General Hospitals |
| Royal Free Hospital, Gray's Inn Road, W.C. | *In- and Out-Physician*, Dr. Hayes, Tu. S. 9 |
| Royal Hospital for Children and Women, Waterloo Bridge Road, S.E. Sec., Mr. R. G. Kestin. | *Out-Physicians*, Daily at 2. *Out-Surgeon*, W. 2. *See* "Children's Diseases." |
| St. Bartholomew's Hospital, Smithfield, E.C. | *In-Physician*, Dr. M. Duncan, Tu. Th. *Out-Physician*, Dr. Godson, W. S. 9 |
| St. George's Hospital, Hyde Park Corner, S.W. | *Physician*, Dr. Champneys, Th. 1.30 |
| St. Mary's Hospital, Cambridge Place, Paddington, W. | *Physicians*, Drs. Meadows, Tu. F. 9.30; H. Jones, M. Th. 1.30 |
| St. Thomas's Hospital, Westminster Bridge Road, S.E. | *Physician*, Dr. Jervis, Tu. F. 2 |
| Samaritan Free Hospital for Women and Children, Lower Seymour Street, W. Branch, 1, Dorset Street, Manchester Square, W. Sec., Mr. Geo. Scudamore. Governor's letter or card is required if the disease of the applicant is *not* peculiar to her sex. Out-patients department (free) is at Edward's Mews, Duke Street, W. | *In-Physicians*, Drs. P. Boulton, M. Prickett, Amand Routh. Daily, 2. *In-Surgeons*, Messrs. G. G. Bantock, J. H. Thornton, W. A. Meredith. Daily, 9.30. *Out-Physicians*, Drs. M. Prickett, and Amand Routh, W. S. 1.30. *Out-Surgeons*, Messrs. W. A. Meredith, and J. D. Malcolm, M. Th.; A. H. G. Doran, and W. S. A. Griffith, Tu. F. Hour 1.30 |
| University College Hospital, Gower Street, W.C. | *Physicians*, Drs. G. Hewitt, M. Th. 1.30; J. Williams, Tu. F. 2 |
| West London Hospital, Hammersmith Road, W. | *Physicians*, Drs. A. Venn, Moullin, W. S. 2 |
| Westminster Hospital, Broad Sanctuary, S.W. | *Physicians*, Drs. Potter, Tu. F. 2; Griggs, Tu. F. 9 |

